# Case report: Ultrasound diagnosis of fish bone penetration into the thyroid

**DOI:** 10.1002/ccr3.2589

**Published:** 2019-12-04

**Authors:** Gaëtan Cavelier, Katharina Ostermann, Mihaela Horoi, Raymond Huvenne, Didier Dequanter, Alexandra Rodriguez

**Affiliations:** ^1^ Department of ENT & Head and Neck Surgery CHU Saint‐Pierre Brussels Belgium; ^2^ Faculty of Medicine Free University of Brussels Brussels Belgium; ^3^ Department of Radiology CHU Saint‐Pierre Brussels Belgium

**Keywords:** fish bone, migration, penetration, thyroid gland, ultrasounds

## Abstract

Ultrasonography is useful in the diagnosis of foreign body migrations (eg, fish bones) into the soft tissues of the neck.

## Graphical abstract

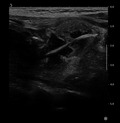

## 1 INTRODUCTION

A common cause of otolaryngology visits is esophageal and pharyngeal foreign bodies. Among these foreign bodies, fish bones appear to be the most prevalent. The usual symptoms include throat pain, odynophagia, and globus. However, only less than 60 cases of extraluminal migration, mostly to the thyroid gland, are found in the literature. The diagnosis is difficult and often delayed because symptoms are unspecific.

## 2 CASE REPORT

**Figure 1:**
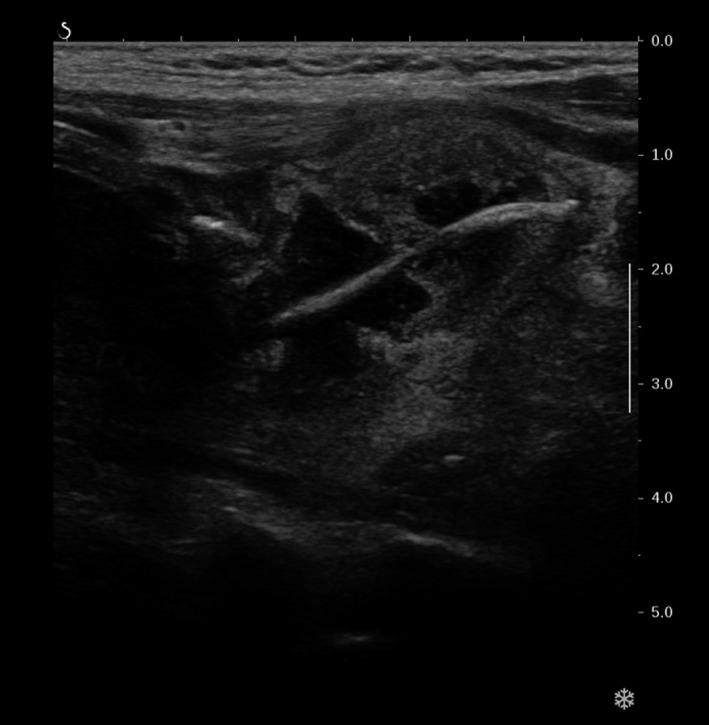
Ultrasonography image showing the fish bone in the left lobe of the thyroid gland surrounded by an abscess

A 59‐year‐old African woman was admitted to our medical department with throat pain, odynophagia, and mild sialorrhea after swallowing a fish bone (red mullet) the previous night. Flexible endoscopy of the pharynx, larynx, and upper esophagus could not identify a foreign body. No mucosal lesion was observed, and the patient was discharged with an antalgic treatment. At the follow‐up appointment 72 hours later, symptoms had worsened with the appearance of cervical swelling and pain. On clinical examination, no foreign body was found. Cervical ultrasonography (US) was conducted and showed a 2‐cm‐long foreign body in the left lobe of the thyroid gland with a surrounding collection. (Figure 1).

The patient was hospitalized and antibiotics started.

A blood sample showed an elevation of inflammatory biomarkers (C‐reactive protein and white blood cells), and thyroid markers were normal except for the thyroglobulin which was elevated to 2540 mcg/L (normal: <77 mcg/L). Further laboratory studies the following day revealed hyperthyroidism. (Table 1).

**Table 1:** Blood samples results

	Reference value range	Units	Day 1	Day 2	Day 15
White blood cells	3.5‐11.0	×10^3^/μL	22.69	23.46	6.57
C‐reactive protein	<10	mg/L	260.8	272.3	2.9
TSH	0.27‐4.20	mU/L	0.45	0.23	—
Thyroglobulin	<77	μg/L	2540	—	—
Free T4	12.0‐22.0	pmol/L	21.8	25.8	—
Free T3	3.10‐6.80	pmol/L	—	7.85	—

Abbreviation: TSH: thyroglobulin‐stimulating hormone.

**Figure 2:**
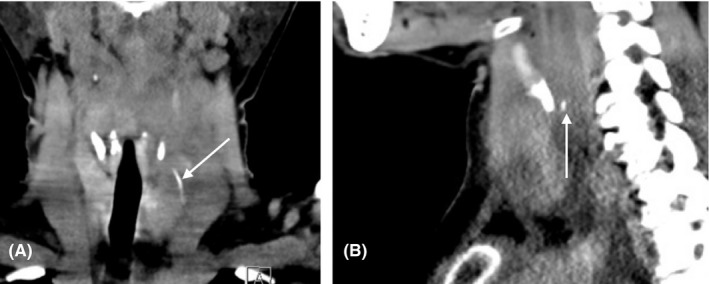
Computed tomography images. A, Coronal image of the fish bone surrounded by an abscess collection. B, Sagittal image of the fish bone. Arrows showing the fish bone

Computed tomography (CT) confirmed the presence of a 2.8‐cm‐long hyperdense body with a surrounding collection and an infiltration of the perithyroid and retropharyngeal fat. (Figure 2) Rigid pharyngolaryngoscopy and esophagoscopy were performed, but neither the entry point nor the fish bone was found.

A left loboisthmectomy was consequently performed following a 15‐day antibiotic window. After resection, the fish bone was palpable in the specimen. The anatomopathological examination confirmed the presence of a birefringent foreign body with a granulomatous reaction. Postoperatively, the initial complaints resolved completely, and thyroid markers were normalized. No adverse events occurred, and the patient was discharged the following day.

## 3 DISCUSSION

Foreign bodies are common in otorhinolaryngology visits and must be taken seriously. Not only can they be inhaled, but there is also a risk of migration into soft tissues of the neck which can cause neurovascular lesions,^1^ development of abscesses, and fistulas.

The initial evaluation should consist of a detailed history including the type of foreign body (eg, fish versus animal bone, wood, and metal) and physical examination (ie, palpation and endoscopy) within the outpatient department. If no foreign body is found, symptoms of pain and globus can also be due to small lesions of the mucosa. Symptomatic treatment should be prescribed. In our opinion, the patient should be evaluated between 48 and 72 hours after the start of treatment to confirm resolution of the symptoms. The persistence of symptoms may indicate the presence of a foreign body which may, for example, be hidden in a mucosal fold or in an extraluminal migration.^2^

In this case, the acute onset leads us to consider it more as a penetration rather than as a slow migration, which distinguishes it from the previously reported cases. Indeed, our patient had eaten a piece of bread after swallowing the fish bone that was immediately associated with strong odynophagia. The mechanical force after swallowing the bread may have pushed the fish bone from the hypopharynx to the thyroid gland.

Several tools are available in clinical practice to make this diagnosis.

Plain radiography is often used since it can identify radiopaque foreign bodies (eg, woods, metal, and animal bones) including some fish bones with a sensitivity of 79%.^3^

CT has a sensitivity of 100% in the soft tissues and can be useful, not only for preoperative planning but also for evaluating complications.^4^

Ultrasound offers the advantages of high sensitivity, low cost, and portability without the risks of radiation. Aras et al showed that ultrasounds were superior to CT and plain radiography for foreign bodies with low radiopacity in the tissues. However, in air, CT is better than ultrasounds.^4^

Pharyngo‐laryngo‐esophagoscopy should be performed even in case of migration because the entry point can sometimes be found and the foreign body removed without requiring an open surgery.^5^ Indeed, Petrarolha et al^6^ were able to find a fish bone and remove it without the need of thyroidectomy. Nevertheless, the patient must be informed that open surgery might be necessary as in our case. Interestingly, another team performed a thyroid cartilage window to remove a foreign body that migrated to the paraglottic space after the failure of rigid laryngoesophagoscopy.^7^

## 4 CONCLUSION

Foreign body migration or penetration into the thyroid gland is rare and difficult to diagnose. When no foreign body can be found with flexible and rigid endoscopy within the outpatient department, ultrasound can be a very useful, low‐cost, and safe tool with high diagnostic sensibility. Nevertheless, computed tomography remains useful, especially for presurgical evaluation. Clinicians should pay close attention to patients with persistent symptoms following a history of acute dysphagia and systematically perform a cervical ultrasound in such cases.

## CONFLICT OF INTEREST

None Declared.

## AUTHOR CONTRIBUTIONS

G. Cavelier, MD: involved in writing and served as assistant surgeon; K. Ostermann, MD: involved in flexible fiberoptic examination and served as paper reviewer; M. Horoi, MD: served as paper reviewer and surgeon; R. Huvenne, MD: served as radiologist; D. Dequanter, MD, PhD: served as cervico‐facial surgeon and paper reviewer; A. Rodriguez, MD: served as cervico‐facial surgeon, rigid laryngo‐pharyngo‐esophagoscopy and paper reviewer.
